# Joint Hypermobility: An Under-Recognised Cause of Palpitations, Dizziness, and Syncope in Young Females

**DOI:** 10.3390/jcm14207373

**Published:** 2025-10-18

**Authors:** Zeina Abu Orabi, Sophie E. Thompson, Jan van Vliet, Kate Gee, Ashwin Roy, Jonathan N. Townend

**Affiliations:** 1 Department of Cardiology, Queen Elizabeth Hospital Birmingham, University Hospitals Birmingham NHS Trust, Birmingham B15 2GW, UK; zeina.abuorabi@nhs.net (Z.A.O.);; 2 Department of Cardiovascular Sciences, School of Medical Sciences, College of Medicine and Health, University of Birmingham, Birmingham B15 2TT, UK; 3 Midland Metropolitan Hospital, Sandwell and West Birmingham NHS Foundation Trust, Birmingham B66 2QT, UK

**Keywords:** joint hypermobility, orthostatic intolerance, Postural orthostatic tachycardia syndrome, Ehlers Danlos syndrome, hypermobility spectrum disorder

## Abstract

**Background:** Symptoms of dizziness, syncope, and palpitations are common presentations in outpatient and emergency care, frequently attributed to stress and anxiety when conventional neurological and cardiac evaluations are normal. Joint hypermobility (JH) syndromes including hypermobile Ehlers–Danlos syndrome (hEDS), and hypermobility spectrum disorders (HSD) are under-recognised as potential causes. **Methods:** Our retrospective cohort study examined the clinical features, diagnostic findings, and responses to treatment in patients with JH syndromes, who are referred to a specialised syncope clinic within a UK teaching hospital. It involved 218 patients with joint hypermobility, predominantly young females (median Beighton score: 6), reporting chronic orthostatic intolerance, dizziness, and palpitations. Common comorbidities included joint pain, chronic fatigue, gastrointestinal dysmotility, and psychiatric conditions. Prevalence of symptoms, cardiovascular abnormalities on investigation (ECG, echocardiography, and tilt-table testing), and treatment responses were analysed. **Results:** History and examination were often diagnostic. Standard cardiac tests rarely provided diagnostic value except to exclude alternate conditions. Tilt-table testing was abnormal in 82.0% of cases, revealing orthostatic hypotension, reflex syncope, or postural tachycardia syndrome (POTS). Conservative measures (hydration, salt intake, and exercise) were effective in over half of the cases; pharmacological treatments (ivabradine, fludrocortisone) were considered for refractory cases. **Conclusions:** This study emphasises that JH syndromes are a common cause of palpitations, dizziness, and syncope in young females. They are multi-system conditions affecting both physical and mental health, which remain under-recognised and are often dismissed as ‘functional’, particularly in women—highlighting gender bias in diagnosis. A structured diagnostic approach with routine joint assessments for JH and increased awareness can facilitate early recognition and management in general medical settings, reducing reliance on emergency services and improving patient outcomes.

## 1. Introduction

Symptoms of dizziness, syncope, and palpitations are common reasons for referral to both outpatient services and the emergency department (ED). Syncope alone is said to account for 1–3% of ED attendances, while palpitations may account for a further 0.5% [1,2]. Frequent approaches involve investigations for epilepsy and/or cardiac evaluations to look for structural abnormalities and arrhythmia, usually involving auscultation, an ECG, ambulatory ECG monitoring, and transthoracic echocardiography (TTE). When no abnormality is apparent, many patients, particularly young adults, are discharged with reassurance that there is ‘no cardiac or neurological abnormality’ along with suggestions of symptoms due to stress and anxiety.

Joint hypermobility (JH) syndromes are well recognised by rheumatologists as causes of joint instability and chronic pain but are often not appreciated by the medical profession as common causes of dizziness, pre-syncope, syncope, and palpitations. While the problem of ‘hypermobility syndrome’ causing joint laxity was first described in 1967, the extra-articular manifestations of orthostatic intolerance together with chronic pain, gastrointestinal dysmotility, psychiatric disease, urinary tract problems, and chronic fatigue were recognised only gradually during the 1980s and 1990s [3]. Why this common problem affecting around 3% of the population and up to 26% of adolescents is so poorly appreciated and understood remains unclear [4,5].

This report describes the experience of a single dedicated syncope clinic based in a large university teaching hospital in the UK. A single physician (JNT) took over the running of this clinic in 2020 and noted that many young adult female patients were being referred by primary care, often following ED attendance, with a constellation of symptoms consisting of dizziness, rapid palpitations, syncope, effort, and orthostatic intolerance. In many cases, the referring doctor questioned a diagnosis of anxiety or postural orthostatic tachycardia syndrome (POTS). Given the number of such patients with a history of JH/hypermobile Ehlers–Danlos Syndrome (hEDS) and the previous reports of an association between JH and POTS, it was decided to look for evidence of JH in all such patients referred to the clinic. Accordingly, from 2020, all new patients with possible POTS (dizziness, palpitations, or syncope) were assessed with a Beighton score in addition to a standard cardiovascular assessment. This report describes the characteristics of a large group of these under-recognised, mainly female patients with chronic disability and aims to characterise their cardiovascular and other symptoms via physical and investigation findings after a uniform diagnostic testing approach.

## 2. Methods

### 2.1. Population and Data Collection

Patients with JH were identified from an electronic register of patients attending the syncope clinic at the Queen Elizabeth Hospital Birmingham between February 2021 and July 2024. Criteria included a Beighton score of >4 along with a typical symptom constellation of orthostatic intolerance, dizziness, and palpitations. A pragmatic approach to the diagnosis of JH was used using a detailed history examination to determine a Beighton score. No consistent attempt was made to differentiate hEDS from HSD (see Section 4). The clinic was run by a consultant cardiologist who documented each patient in an initial clinic letter and arranged a standard set of investigations. A retrospective review of the hospital’s electronic patient record system was conducted at the beginning of August 2024 to review clinic letters, where data on patient-reported past medical history, medication history, and social history were gathered. Data were also collected on the number of ED attendances and the reason for presentation. Clinical observations were recorded, including blood pressure, pulse rate, height, and weight, as well as the results of initial investigations, which in most cases included TTE, 24 h ambulatory ECG, and a tilt-table test.

### 2.2. Diagnostics

All investigations were performed according to standard departmental protocols. TTE was performed by accredited technicians according to the British Society of Echocardiography’s minimum dataset [6] and analysed using IntelliSpace Cardiovascular (Philips, Amsterdam, The Netherlands). Ambulatory ECG monitoring was most commonly undertaken for a 24 h period; however, some patients underwent a longer period of monitoring (up to 72 h). Tilt-table testing was performed according to a standard protocol (40 min stand at 70 degrees), without drug provocation [7]. Definitions of abnormalities on tilt-table testing are described in Table 1.

### 2.3. Statistical Analysis

Data analysis was performed using SPSS (SPSS Inc., Chicago, IL, USA; 28.0.1). Descriptive statistics, frequencies, and percentages were generated. Data are presented as the mean (standard deviation) or median (interquartile range).

### 2.4. Ethical Considerations

This study was approved by the Clinical Audit Department at the Queen Elizabeth Hospital Birmingham (CARMS: 19486 and date of approval: 24 July 2023). In accordance with the UK National Research Ethics Service guidance, neither individual informed consent nor a formal research ethics committee review was required as the study was undertaken by the direct clinical team using data previously gathered during the course of routine care and sufficiently anonymised.

## 3. Results

### 3.1. Demographics

A total of 218 patients with JH attended the syncope clinic between February 2021 and July 2024, with a median follow-up period of 1.2 years (an IQR of 2.2 years; a range of 2 months to 4.7 years). Most patients were female (95.4%) and of white ethnicity (65.1%), with a median age of 24 years (IQR 11.0). The mean BMI of the patients was 26.5 (SD 6.6), though 44.0% of patients were categorised as overweight or obese. Almost one in three (29.4%) patients with JH were not in employment, education, or training. The median Beighton score at the first clinic visit was six (an IQR of three). Although the majority of patients (59.2%) were mildly or moderately limited in terms of mobility by their condition, 18 (8.3%) reported being severely limited, while 6 patients (2.8%) were wheelchair-bound. Psychiatric disease was common, affecting 67.9% of the patients, principally depression (22.9%), anxiety (26.6%), and the combination (13.3%). Almost half (45.9%) of the patients were on psycho-active medication. ADHD and autism affected 7.8% and 7.3% of the patients, respectively. Other common comorbidities included migraine (34.9%), asthma (14.2%), mast cell activation/allergy syndrome (12.4%) and fibromyalgia (6.9%). Baseline characteristics are summarised in Table 2.

### 3.2. Symptoms

Patients had been experiencing symptoms for a median of 5.0 years (an IQR of 7.0) prior to their first visit to the syncope clinic. The predominant cardiovascular symptom was dizziness upon standing (77.1%), followed by syncope (10.1%) and palpitations (9.6%). Although not the predominant symptom in most, palpitations were common as a secondary symptom, affecting around half of the patients (50.9%). Additionally, non-cardiovascular symptoms were highly prevalent. The commonest were gastrointestinal (40.4%), migraine (34.9%), chronic pain (34.4%), joint pain/dislocation (28.0%), and chronic fatigue (25.2%). ED attendance data was available from 2018 onwards. Over a 6-year period (2018–2024), 136 of these patients attended ED at trust hospitals at least once. In total there were 861 ED attendances between 136 patients. Cardiovascular complaints were a common reason for emergency presentation, with 116 attendances due to non-specific chest pain, 45 with shortness of breath, 45 with dizziness or pre-syncope, 25 with palpitations, and 12 with syncope. Almost one-third of those admitted were classified as having seizures (n = 46).

### 3.3. Cardiovascular Investigations

#### 3.3.1. Baseline Physiological Parameters

The mean heart rate of the patients not on rate-limiting medications in the clinic was 99 beats per minute (bpm) (an SD of 17.1). In those taking rate-limiting medications already (n = 7), the clinic heart rate was 81 bpm (an SD of 13.0). The mean systolic blood pressure in the clinic was 125 mmHg (an SD of 13.9), and the mean diastolic blood pressure was 80 mmHg (an SD of 8.5). A minority of patients were identified as having postural hypotension in the clinic, with a fall in systolic BP of > 20 mmHg upon standing (10.6%).

#### 3.3.2. Electrocardiography and Ambulatory Monitoring

**Figure 1:**
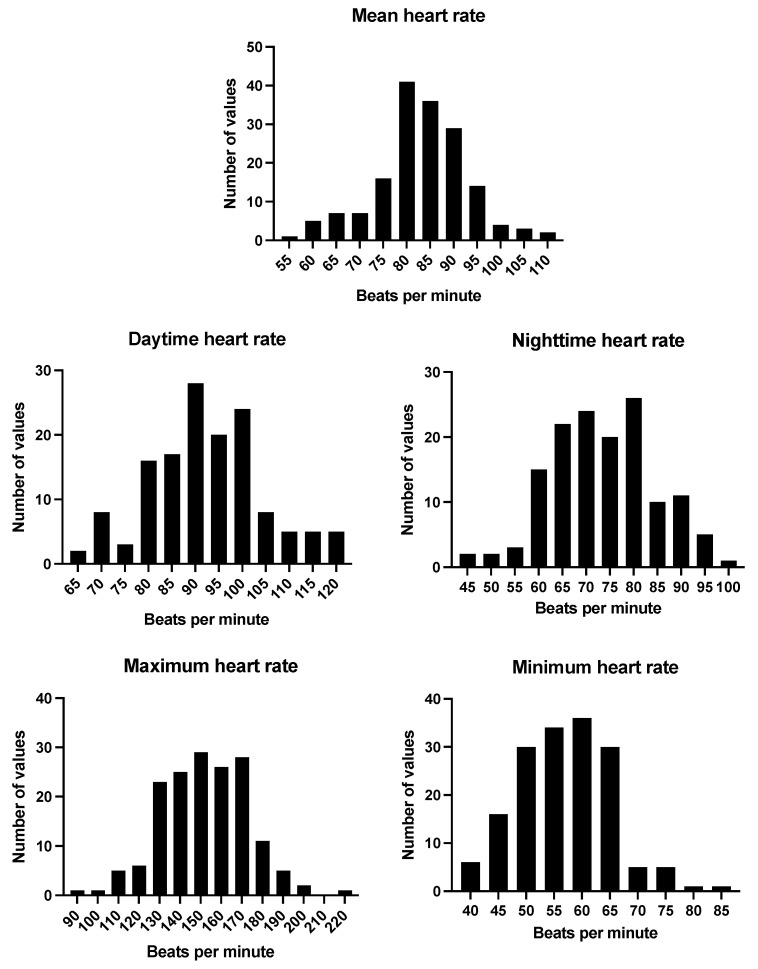
Histograms of ambulatory heart rate data.

All patients had a baseline ECG. This was normal in most (76.1%). Sinus tachycardia (heart rate (HR) > 100 bpm) was identified in 27 patients (12.4%), while sinus bradycardia was seen in 2 patients (0.9%). ST/T wave changes were identified in 23 patients (10.6%). Other notable ECG findings detected included a long QT interval (n = 1), right-axis deviation (n = 2), and intraventricular conduction delay (right bundle branch block (RBBB) or incomplete RBBB, n = 2). Of the 165 patients who had complete ambulatory ECG monitoring, the average 24 h HR of patients off rate-limiting medications was 83 bpm (an SD of 9.9). All patients showed a physiological fall in nocturnal heart rate; the mean daytime HR was 92 bpm (SD 12.2), and the nighttime HR was 74 bpm (SD 10.9). No significant arrhythmias (other than sinus tachycardia) were detected amongst the cohort on Holter monitoring. The ECG findings and heart rate data are summarised in Table 3 and Figure 1.

#### 3.3.3. Transthoracic Echocardiography

TTE was performed in 198 patients, of which the majority were normal (83.9%). Five patients (2.3%) were noted to have elongation of the anterior mitral valve leaflet, and three (1.4%) had bileaflet bowing of the mitral valve without significant prolapse. Seven patients (3.2%) had mild valvular regurgitation (mitral in three, tricuspid in two, aortic in one, and pulmonary in one). Incidental findings included a small pericardial effusion and a small perimembranous ventricular septal defect (VSD). One patient was already known to have mild–moderate aortic stenosis under follow-up elsewhere. None had significant aortic root dilatation.

#### 3.3.4. Tilt-Table Testing

**Figure 2:**
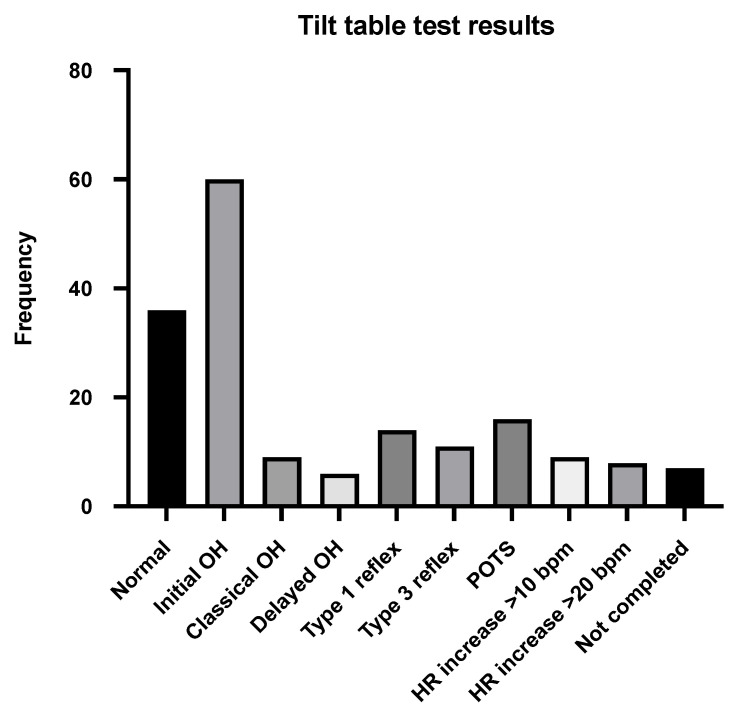
Tilt-table test findings.

Around three-quarters of patients had a tilt-table test (n = 162, 74.3%). This was normal in just 36 patients (16.5%), including 3 who had symptoms during the test. The test was not fully completed in seven patients (3.2%). Initial orthostatic hypotension (OH) was the most common abnormality during tilt-table testing, as identified in 60 (27.5%) patients. Nine patients (4.1%) had classical OH, while six (2.8%) had delayed OH. Type 1 mixed reflex syncope was seen in 14 patients (6.4%), and type 3 vasodepressor reflex syncope was seen in 11 patients (5.0%). A group of patients demonstrated abnormal heart rate responses. Sixteen patients (7.3%) met the diagnostic criteria for POTS, with a sustained increase in heart rate within 10 min of tilt of >30 bpm. Nine patients (4.1%) displayed an increase in heart rate of >10 bpm within 10 min, and eight patients (3.7%) showed an increase in heart rate of >20 bpm within 10 min. (Table 3 and Figure 2).

### 3.4. Response to Management

At the time of the first clinic visit, 52 patients (23.9%) were already implementing conservative measures such as increased fluid (2–3 L daily) and salt intake. Over half of the patients (n = 127, 58.3%) responded favourably to conservative measures. Some patients had already been initiated on medical treatment prior to their first clinic visit, including 26 (11.9%) on a beta-blocker, 21 (9.6%) on ivabradine, and 5 (2.3%) on fludrocortisone. Medical management in the syncope clinic included introduction of a new medication or dose increase and was initiated in 128 (58.7%) patients. The dose of an existing cardiovascular medication was increased in 14 patients (6.4%). Overall, 56 (43.8%) patients reported complete symptomatic responses to medical therapy. Over a quarter of patients (n = 36, 28.1%) reported some improvement in their symptoms with medical management. Only 8 (6.3%) patients did not respond to medical therapy, and 28 (21.9%) have not yet been followed up to assess their response (Table 4).

## 4. Discussion

This retrospective analysis from a single UK hospital demonstrates that there are many young female patients with JH syndromes and long-standing, disabling cardiovascular symptoms for which no cause has been identified and for whom no effective treatment has been provided. Most have been in frequent medical contact in primary and secondary care. This report illustrates the importance of a thorough history and routine rapid joint assessment using the Beighton score to reveal the diagnosis. This adds little time or expense to the consultation. We also provide new information on the low prevalence of echocardiographic abnormal findings and on the high prevalence of abnormalities from investigations by ambulatory monitoring and tilt testing in patients with JH. Our report draws attention to additional physical problems related to JH including joint pain, chronic fatigue, chronic pain, gastrointestinal and urinary problems, and migraine, which were all highly prevalent. Lastly, psychological problems and disorders including anxiety and depression, with autism and ADHD being heavily over-represented, a finding consistent with previous reports of a strong association between JH syndromes and autistic spectrum disorder [8,9]. The association of POTS and orthostatic intolerance with JH syndromes is not a new finding [10,11], but our study demonstrates the high prevalence of this disorder in young women presenting with symptoms suggestive of POTS and the substantial co-morbidity that is often present.

Considerable and understandable confusion surrounds the precise diagnosis and classification of JH syndromes, with frequent changes in terminology. Most recently, in 2017, the previous term ‘joint hypermobility syndrome’ was renamed as ‘hypermobility spectrum disorders’ (HSD) [12]. In addition, type III Ehlers–Danlos syndrome was renamed ‘hypermobile Ehlers–Danlos syndrome (hEDS)’ and given a new set of diagnostic criteria to differentiate it from HSD. By definition, hEDS is currently genotype-negative, and the criteria are clinical. They involve evidence of JH assessed using the Beighton score; dermatological, skeletal, dental, valvular, or aortic abnormalities (including at least mild mitral valve prolapse or aortic root dilatation); or a family history of chronic pain or recurrent dislocation and exclusion of other connective tissue disorders [13]. If significant hypermobility is present on the Beighton score but a patient does not fit the criteria for hEDS or another of the EDSs, the patient is diagnosed as HSD. The two disorders are difficult to clinically differentiate (indeed, they are widely considered as the same disorder), and as both are associated with the same symptoms and management strategies, there seems little practical reason in attempting to differentiate them in the clinical setting.

Our data demonstrates that standard cardiological investigations for JH syndromes are most helpful to exclude alternative underlying causes. Ambulatory ECG monitoring excluded arrhythmia but showed no diagnostic features other than frequent daytime sinus tachycardia. All patients had a normal fall in heart rate at night. TTE identified a small number of patients with mitral valve abnormalities of low clinical significance. There is a weak association between valvular abnormalities and hEDS, and this is even weaker for HSD. A study of 258 patients by Rashed et al. found that mitral valve prolapse (7.5%) and thoracic aortic root dilatation (15.2%) were common in both hEDS and HSD, although aortic dilatation was more prevalent in those meeting the diagnostic criteria for hEDS (20.7%) than HSD (7.7%) [14]. Additionally, five patients experienced extra-aortic complications including spontaneous carotid artery dissection and coronary artery dissection [10]. A study of a similar size by Asher et al. noted a similar prevalence of mitral valve prolapse (6.4%) and 1.6% with aortic root dilatation [15]. For context, the prevalence of mitral valve prolapse in the general population is estimated at 2.6% [16], while aortic dilatation is estimated at 1.4–4.0% [17,18]. Given the low prevalence of abnormalities and the fact that most were minor findings requiring no intervention, there is a valid argument against the routine use of TTE in these patients unless clinically indicated.

Tilt-table testing was arguably the most useful investigation; however, only 9.9% met the diagnostic criteria for POTS, i.e., a rise in heart rate of more than 30 bpm with symptoms. The frequent presence of resting tachycardia, however, meant that although tachycardia within 10 min of head up tilt was common, the incremental change in heart rate was usually well under 30 bpm. The test did however show a high rate of other abnormalities including initial and late OH and reflex syncope. Identification of autonomic dysfunction helped guide management. Initial management focused on conservative measures such as wearing supportive activewear, increasing water intake to 2–3 L daily, increasing salt intake (>5 g/day), and engaging in low-impact physical activities such as Pilates to improve fitness and core stability. Conservative measures proved effective in over half of the patients. For patients who did not respond adequately, pharmacological treatments were considered. In those who demonstrated inappropriate tachycardia or an abnormal tachycardic response upon tilt-table testing, ivabradine was often offered. Ivabradine, a selective IF channel blocker, has been evaluated in very few small, randomised trials for inappropriate sinus tachycardia and POTS, with results indicating improvements in symptom burden, functional ability, and quality of life [19,20]. Patients with objective OH identified through tilt-table testing and persistent dizziness despite salt and water loading were offered fludrocortisone or midodrine if fludrocortisone was not tolerated or ineffective. We acknowledge that limited data exist on the use of these medications in younger populations and that robust safety data for use during pregnancy is lacking, an important consideration given the predominance of women of childbearing age. Most patients in our cohort experienced complete resolution of their symptoms, demonstrating the effectiveness of this stepwise approach.

Cardiovascular autonomic features in hypermobility syndromes were first described in 1999 and further characterised in 2003 when a study of 48 patients with ‘joint hypermobility syndrome’ found symptomatic OH, POTS, and uncategorized orthostatic intolerance in 78% of patients versus 10% of controls [21,22]. The mean age at presentation of 24 years in our study is consistent with previous reports [23]. Using objective tests in patients and controls, greater blood pressure responses to a cold pressor test and evidence of pharmacological α-adrenergic and β-adrenergic hyperresponsiveness were demonstrated [18]. Other studies have noted an association between POTS and JH, and it has been estimated that over half with POTS have generalised joint laxity, although our experience would suggest that this is an underestimate [24]. Despite these publications, which are often from over 20 years ago, knowledge about the association of disorders involving cardiovascular autonomic function and JH is not widespread amongst physicians, including cardiologists. In patients without known JH presenting with cardiovascular symptoms, the diagnosis is often not considered and joints are not examined.

The mechanisms underlying the multi-organ symptoms of JH remain poorly understood. An attractive but unproven theory attributes OH and tachycardia to excessive blood pooling in hyper-elastic peripheral vessels, compensatory tachycardia, and reduced blood pressure. Others suggest a ‘dysautonomia’ in which an increased sympathetic tone affects the heart only, with a diminished sympathetic response at the vascular level, limiting the ability to increase blood pressure in response to stimuli such as tilting [25,26]. Small fibre peripheral neuropathy, affecting sympathetic fibres, has also been described [27]. Other mechanisms reported in the literature include adrenergic hyper-responsiveness [28], high circulating levels of histamine and mast cell activation [29], and an association with Arnold Chiari malformation, which can be associated with impaired cardiac autonomic control and resolved following decompressive surgery [30,31]. These diverse mechanisms with a paucity of good-quality supportive evidence highlight the need for further research into the pathophysiology of JH to enable appropriate specific therapies to be developed.

Despite published reports and perhaps because of a lack of clarity on mechanisms of the disease, levels of scepticism about JH syndromes and the contribution of physiological abnormalities to the cardiovascular symptom burden are high, and this may be an example of gender differences in symptoms and/or bias in diagnosis [32]. Women remain under-represented in cardiovascular research and suffer from worse cardiovascular outcomes [33,34]. Studies show that women with cardiovascular conditions are more likely to have their symptoms misattributed to anxiety, delaying appropriate diagnosis and management [35,36]. Our findings would support this. It is unclear whether the mental health problems, including anxiety and ‘hypervigilance’ reported in patients with JH, are an intrinsic part of the disorder related to autism [8,9] or are largely a result of chronic stress related to pain and other disabling symptoms with no effective medical recognition or management. Structural and functional brain abnormalities have been reported [37].

This study is limited by the retrospective nature of data collection, relying on electronic medical records including patient-reported medical and social histories. Although efforts were made to address missing data, this was not always possible. Additionally, this study focused solely on patients with JH referred to the syncope clinic, potentially overestimating the prevalence of cardiovascular symptoms in the broader JH population and reducing the apparent prevalence of non-cardiovascular symptoms. No external validation of the JH diagnosis was made, but there is no available genetic or other biomarker for HSD or h-EDS. We highlight the utility of specialised investigations such as tilt-table testing but acknowledge that such resources may not be readily available in all centres.

## Figures and Tables

**Table 1:** Definitions of responses to tilt-table testing.

Initial Orthostatic Hypotension	Decrease in BP > 40 mmhg at Standing with Fast Normalisation so Symptoms Last < 30 s
Classical orthostatic hypotension	Decrease in systolic BP ≥ 20 mmHg and diastolic BP ≥ 10 mmHg during the first 3 min after standing.
Delayed orthostatic hypotension	Slow and progressive systolic BP decline after the 3rd minute of standing.
Type 1 mixed reflex syncope	HR decreases during syncope but does not reach < 40 bpm or reaches < 40 bpm for <10 s. BP decreases before HR falls.
Type 2 cardioinhibitory syncope	HR decreases < 40 bpm for >10 s. BP decreases before HR falls.
Type 3 vasodepressor syncope	BP falls < 60 mmHg; HR does not fall by more than 10% of the peak value.
Postural orthostatic tachycardia syndrome (POTS)	The increase in HR > 30 bpm or HR > 120 bpm after standing is accompanied by symptoms and BP variability.

Adapted from Practical Instructions for the 2018 ESC Guidelines for the diagnosis and management of syncope|European Heart Journal|Oxford Academic (accessed on 16 October 25).

**Table 2:** Baseline characteristics.

Demographics	n = 218
Age (median, IQR)	24 (11)
Female sex	208 (95.4)
**Ethnicity**	
White British	143 (65.6)
Asian	7 (3.2)
Black	7 (3.2)
Mixed	5 (2.3)
Not reported	56 (25.7)
Body mass index (mean, SD)	26.5 (6.6)
**Body mass index category**	
Underweight	14 (6.4)
Normal	105 (48.2)
Overweight	36 (16.5)
Obese	60 (27.5)
Not reported	3 (1.4)
**Mobility status:**	
No limitation	129 (59.2)
Severe limitation	18 (8.3)
Unable to stand, use of wheelchair	6 (2.8)
**Social history**	
Smoker	19 (8.7)
Alcohol	63 (28.9)
**Employment**	
No work	64 (29.4)
Non-skilled or administrative	33 (15.1)
Skilled or professional	59
Student	53 (24.3)
Unknown	9 (4.1)
**Medication history (pre-clinic)**	
Ivabradine	21 (9.6)
Beta-blocker	26 (11.9)
Fludrocortisone	5 (2.3)
Other *	6 (2.8)
**Main cardiovascular symptom**	
Dizziness on standing	168 (77.1)
Dizziness (any time)	5 (2.3)
Dizziness (exertion)	1 (0.5)
Syncope	22 (10.1)
Palpitations	21 (9.6)
Other (tachycardia)	1 (0.5)
**Non-cardiovascular symptoms**	
Chronic pain	75 (34.4)
Chronic fatigue	55 (25.2)
Joint pain/dislocation	61 (28.0)
Gastrointestinal symptoms	88 (40.4)
Urinary symptoms	17 (7.8)
Migraine	76 (34.9)
Other neurological symptoms (brain fog and blurred vision)	5 (2.3)
**Concomitant disease**	
Psychiatric	
Depression	50 (22.9)
Anxiety	58 (26.6)
Eating disorder	7 (3.2)
ADHD	17 (7.8)
Autism	16 (7.3)
Neurological:	
Migraine	76 (34.9)
Chiari malformation	6 (2.8)
Epilepsy	5 (2.3)
Non-epileptiform attack disorder	6 (2.8)
Rheumatological:	
Rheumatological diagnosis (SLE, connective tissue disease)	8 (3.7)
Raynaud’s phenomena	5 (2.3)
Mast cell activation/allergy syndrome	27 (12.4)
Fibromyalgia	15 (6.9)
Gynaecological	
Endometriosis	4 (1.8)
PCOS	5 (2.3)
Other:	
Asthma	31 (14.2)
Autoimmune disease	6 (2.8)
Inflammatory bowel disease	4 (1.8)

Values presented are n (%) unless otherwise stated. * Nifedipine (n = 2), perindopril (n = 1), ramipril (n = 1), candesartan (n = 1), and verapamil (n = 1).

**Table 3:** Cardiovascular physiology and investigations.

Vital Signs	n = 218
Heart rate off rate-limiting medications (mean, SD)	98.7 (17.1)
Heart rate on rate-limiting medications (mean, SD)	80.6 (13.0)
Systolic BP in clinic (mean, SD)	125.4 (13.9)
Diastolic BP in clinic (mean, SD)	79.8 (8.5)
Postural hypotension in clinic	
Yes	23 (10.6)
No	148 (67.9)
Not performed	47 (21.6)
**ECG**	
Normal	166 (76.1)
Sinus tachycardia	27 (12.4)
Sinus bradycardia	2 (0.9)
Non-specific ST/T wave changes	23 (10.6)
Long QT	1 (0.5)
RBBB/incomplete RBBB	3 (1.4)
Right axis deviation	2 (0.9)
**Echocardiogram**	
Normal	183 (83.9)
Abnormal	15 (6.9)
Not performed	20 (9.2)
**Echocardiogram abnormalities**	n = 15
Mild valvular regurgitation	7 (3.2)
Elongated AVML	5 (2.3)
Bileaflet bowing of MV	3 (1.4)
Other incidental finding *	3 (1.4)
**Ambulatory ECG monitoring** (off rate-limiting medications)	n = 165
Mean HR in bpm (mean, SD)	83 (9.9)
Max HR in bpm (mean, SD)	152 (21.0)
Min HR in bpm (mean, SD)	57 (8.4)
Mean daytime HR in bpm (mean, SD)	92 (12.2)
Mean nighttime HR in bpm (mean, SD)	74 (10.9)
Tilt-table test **	n = 162
Normal	36 (22.2)
Initial OH	60 (37.0)
Classical OH	9 (5.6)
Delayed OH	6 (3.7)
Type 1 mixed reflex syncope	14 (8.6)
Type 3 vasodepressor reflex syncope	11 (6.8)
POTS	16 (9.9)
HR increase > 10 bpm within 10 min	9 (5.6)
HR increase > 20 bpm within 10 min	8 (4.9)
Test not completed	7 (4.3)

Values presented are n (%) unless otherwise stated. * Other incidental findings include pericardial effusion (n = 1), perimembranous VSD (n = 1), and known mild–moderate aortic stenosis (n = 1). ** Some had more than 1 abnormality.

**Table 4:** Summary of management.

	n = 218
**Existing management**	
Already implementing conservative measures	52 (23.9)
Already taking rate limiting medications	47 (21.6)
**Conservative management**	
Response to conservative management	
Yes	127 (58.3)
No	61 (28.0)
Not reported or not yet followed up	30 (13.8)
**Medical management**	
New medication initiated	
Ivabradine	63 (28.9)
Beta-blocker	17 (7.8)
Fludrocortisone	39 (17.9)
Midodrine	20 (9.2)
Dose of existing medication increased	14 (6.4)
Response to medical management (n = 128)	
Complete response	56 (43.8)
Some response	36 (28.1)
No response	8 (6.3)
Not yet followed up or not reported	28 (21.9)

Values presented are n (%) unless otherwise stated.

## Data Availability

The data underlying this article were obtained from electronic medical records at the Queen Elizabeth Hospital Birmingham and are subject to privacy and ethical restrictions. The source data are therefore not publicly available, but anonymized data are available, subject to reasonable request.
